# The Examination of the Influence of Caffeinated Coffee Consumption on the Concentrations of Serum Prolactin and Selected Parameters of the Oxidative-Antioxidant Balance in Young Adults: A Preliminary Report

**DOI:** 10.1155/2022/1735204

**Published:** 2022-07-25

**Authors:** Kamil Rodak, Izabela Kokot, Aleksandra Kryla, Ewa Maria Kratz

**Affiliations:** Department of Laboratory Diagnostics, Division of Laboratory Diagnostics, Faculty of Pharmacy, Wroclaw Medical University, Borowska Street 211A, 50-556 Wroclaw, Poland

## Abstract

We verified whether caffeinated coffee consumption influenced the concentrations of prolactin (PRL) and oxidative stress parameters: total antioxidant status (TAS), ferric reducing antioxidant power (FRAP), total oxidant status (TOS), oxidative stress index (OSI), advanced oxidation protein products (AOPP), uric acid (UA), total bilirubin (T-Bil), albumin (ALB), iron (Fe), calcium (Ca), magnesium (Mg), and inflammatory marker C-reactive protein (CRP)—in blood sera obtained at 15, 60, and 120 minutes after caffeinated coffee intake, in relation to the fasting point. The study participants were 33 young, healthy, nonsmoking volunteers (15 men, 18 women) aged 19-29 years. PRL concentrations significantly decreased (*p* < 0.05) after consumption, except at time point 15' in men (*p* > 0.05). In women, FRAP levels significantly increased over time, and significant changes were also observed for UA at 120' and ALB at 15'. In men, significant changes were found for levels of AOPP at 15', T-Bil and ALB at 15', iron at 60' and 120', and calcium at 120'. There were no significant differences in the levels of other examined parameters between the defined time points. In conclusion, the substances contained in caffeinated coffee decrease the level of prolactin and may also have an impact on selected parameters of oxidative stress, which could be the basis of future research focused on the identification of new therapeutic targets.

## 1. Introduction

**Figure 1:**
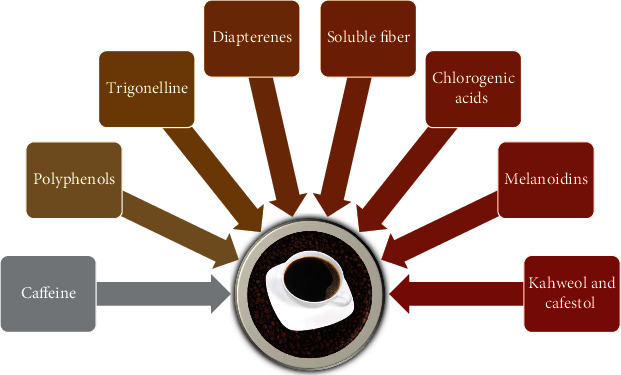
The main ingredients of coffee with an antioxidant effect.

Coffee is the most popular stimulant among people all around the world. Its main active compound is caffeine (1,3,7-trimethylxanthine) [1], which has both positive (e.g., better concentration, higher agitation, reduced risk of Alzheimer's Disease and Parkinson's Disease development, and anti-inflammatory properties) and negative (e.g., increased risk of lung cancer, anxiety, and urinary incontinence) effects on body functioning [2]. Also, caffeine is considered to have antioxidant properties, as evidenced by the affinity for scavenging hydroxyl radicals [3]. Ninety-nine percent of caffeine is absorbed from the gastrointestinal tract within 45 minutes of consumption of caffeinated beverages, and the maximum blood plasma concentration of this compound is observed 15–120 minutes after oral intake [4]. However, it is worth remembering that coffee contains not only caffeine but is composed of many bioactive compounds with an anti-free-radical or antioxidant effect, including phenolic compounds (e.g., tocopherols), trigonelline, diterpenes, soluble fiber, chlorogenic acids (CGAs), cafestol, and kahweol in various quantities, depending on the source [5–8]. The total content of polyphenols ranges from 200 to 550 mg per cup [9]. Another important contribution may be the action of some compounds generated during the thermal reactions of the roasting process, such as melanoidins, which show strong antioxidant properties [10]. The main antioxidant substances present in coffee are shown in Figure 1. The effects of coffee on the human organism depend mainly on its type, quantity, the blend of coffee (i.e., Arabica or Robusta), the growing area, the extent of roasting, the method of brewing [11], and the age and sex of the person who drinks it. The observed changes are mainly caused by the interaction of its ingredients with receptors such as adenosine receptors [2]. For example, Gorjanović et al. [12] compared 24 different types of coffee and showed that instant coffees contain the highest amount of antioxidants because they are the richest in polyphenols out of all coffee types. Moreover, Czachor et al. [13] compared Robusta and Arabica and showed that Robusta had a greater amount of caffeine, polyphenols, and antioxidant activity.

For decades, scientists have debated the effects of coffee on human health. The controversy surrounding this topic even led to the fact that in 1991 the International Agency for Research on Cancer (IARC) classified coffee as “possibly carcinogenic to humans” because of a weak positive relationship between coffee consumption and the risk of bladder, pancreatic, and ovarian cancer [14]. Recently, caffeinated coffee became a subject of interest for scientists because of its antioxidant properties [6, 9, 15, 16]. Long-term consumption of coffee and caffeine has been shown to play an important role in preventing age-related cognitive decline by protecting the antioxidant system and regulating oxidative stress [17]. A study conducted by Qureshi et al. [18] documented that coffee ranked in the top places among drinks contributing the most to the total antioxidant intake through dietary habits in women. More and more substances of natural origin were tested for their antioxidant and anti-inflammatory properties. One of the most recent reports in this field is the work of Taysi et al. [19], which noted that thymoquinone derived from the herb *Nigella sativa*, commonly used in alternative medicine, exhibits the above-mentioned healing properties. Studies carried out in recent decades confirmed that excessive accumulation in body fluids of reactive oxygen species (ROS), such as the superoxide anion, hydroxyl radical, and hydroperoxyl radical, is a major cause of oxidative stress (OS) and consequently leads to pathological changes in the human body, resulting in premature aging and many diseases. Excessive formation of reactive nitrogen species (RNS) is also among the factors that can trigger oxidative stress and cause nitrosative stress [20]. Oxidative/nitrosative stress occurs when the cellular production of ROS/RNS exceeds the availability of human antioxidants able to defeat these insults. Prolonged oxidative stress may lead to macromolecular oxidative damage, induce tissue protein denaturation, DNA damage, and lipid peroxidation, and interfere with the body's normal metabolic activity, leading to dangerous diseases such as cancer, cardiovascular diseases, or diabetes. In addition, ROS can induce platelet adhesion and aggregation, leading to intravascular coagulopathy, which causes placental infarction and impairs the uteroplacental blood flow, which may consequently lead to deficiencies in oxygen and nutrients necessary for normal fetal development, and thus, oxidative stress can also negatively influence the course of pregnancy [21]. ROS can be produced as a response to various negative factors, such as gamma or UV radiation [22], smoking [23], alcoholism [24], environmental factors [25], polluted and poor-quality food [26], stress [27], some medications or treatments [28], and elements deficiencies (e.g., zinc) [29]. Since higher prolactin levels have also been documented to induce oxidative stress and damage [30], PRL was in our area of interest in the current research.

Prolactin (PRL) is a protein hormone produced by the pituitary gland and has over 300 described functions, including regulation of reproductive function, the immune system, osmotic balance, and angiogenesis. Although the concentration of PRL in body fluids has seemed important only for women, the latest research suggests that PRL is involved in reproduction processes in both women and men, which is of great importance, especially in the case of people of reproductive age. In women, its concentration is related to the onset of ovulation, while the mechanisms of its effects on male fertility remain unclear [31]. Some animal studies reported its role in spermatogenesis [32]. Low PRL levels have also been associated with reduced volume of ejaculate and dysfunction of seminal vesicles in infertile individuals. Moreover, in men, lower PRL levels were associated with erectile dysfunction and premature ejaculation, as confirmed in the general European population and in infertile men [33]. Studies conducted on human sperm have suggested that PRL contributes to the survival of male germ cells, because after incubation with PRL, their mobility was maintained for a longer time, and the spontaneous fragmentation of DNA strands was reduced [34]. Sex hormones, including prolactin, have also been implicated in the etiology of breast and ovarian cancer [31]. Prolactin excess (hyperprolactinemia) may lead to hypogonadism, galactorrhea, etc. In addition to physiological causes such as ovulation and pregnancy, hyperprolactinemia may also be caused by prolactin-secreting pituitary adenoma, liver cirrhosis, polycystic ovarian syndrome, and stress, which may be related to oxidative stress [35]. Due to the widespread consumption of caffeinated coffee by people of reproductive age and the key role of prolactin in processes related to female and male fertility, it seems important to determine the influence of the commonly known stimulant, coffee, on the concentration of this hormone. Some studies emphasized the effects of coffee on levels of sex hormones in the human circulatory system [36–38]. Data from these studies suggest that caffeine and caffeinated coffee may alter levels of circulating luteal estrogens and prolactin, which are possible mechanisms by which caffeinated coffee or caffeine may be associated with malignancies in the reproductive system as well as with fertility disorders.

Currently, measurements of oxidative-antioxidant balance in the human body are based, among others, on the determination of oxidative stress markers such as total antioxidant capacity (TAC), which can be measured by various methods such as total antioxidant status (TAS) and ferric reducing antioxidant power (FRAP), total oxidant status (TOS), and advanced oxidation protein products (AOPP). TOS is usually used to estimate the body's overall oxidation status [39], while the antioxidant capacity of TAS and FRAP is measured using single electron transfer (SET) analytical methods, in which the reducing capacity toward any molecule by electron donation is measured [40].

Apart from the parameters determining the overall oxidative or antioxidant status, single parameters assessing the influence of oxidative stress, such as AOPP, can be analyzed. Due to the high amount of total proteins in human serum and their potential to scavenge ROS, especially by albumin, AOPP appears to be a good marker of oxidative damage induced in the body. As they are factors long circulating in the blood, arising in response to oxidative stress, they can be determined for many hours or even days after the activation of human neutrophil and monocyte oxidative metabolism [41].

The aims of our study focused on the effects of caffeinated coffee consumption on levels of serum prolactin and selected parameters of the oxidative-antioxidant balance of the human body. The objective of the present preliminary study was to assess the short-term effects of caffeinated coffee intake on levels of prolactin and selected parameters of oxidative stress—TAS, FRAP, TOS, AOPP, uric acid (UA), total bilirubin (T-Bil), albumin (ALB), and iron (Fe)—in human serum. We also were interested in the impact of caffeinated coffee on levels of some other elements such as calcium (Ca), magnesium (Mg), and inflammatory markers: C-reactive protein (CRP), which, together with white blood cell (WBC) count, may also be used to verify the health status of the volunteers who participated in our study.

## 2. Materials and Methods

### 2.1. Participants

We enrolled 33 healthy, young adults from Wroclaw Medical University (aged 19-29 years; 15 men and 18 women) in this study. All participants gave written informed consent after being fully informed of the study's aims and procedures. The participants were qualified for the study based on the initial screening questions. The exclusion criteria were a history of hyperprolactinemia, chronic disease, past cancer, inflammation, or cigarette smoking, as reported by the participants. Afterward, an extensive interview included information such as age, gender, medical history, drugs, sports condition, diet, coffee and caffeine consumption, and general health status. All participants were free from any known immune, cardiovascular, metabolic diseases, or illnesses and were not taking any medication. They were all habitual caffeine consumers (from brewed coffee, espresso coffee, instant coffee, or tea). Volunteers included in the project assessed their health as good on the day of the examination and in the preceding two weeks. Due to procedures related to the COVID-19 pandemic at Wroclaw Medical University, each person's body temperature was verified (the acceptable measurement was <37.4°C). Moreover, in fasting samples (time point 0'), we verified the basic parameters of inflammation (WBC < 10 G/L and CRP < 10 mg/L), based on which we made the final decision to include the participants in the study group. The characteristics of the analyzed groups are presented in Table 1.

### 2.2. Study Design

**Figure 2:**
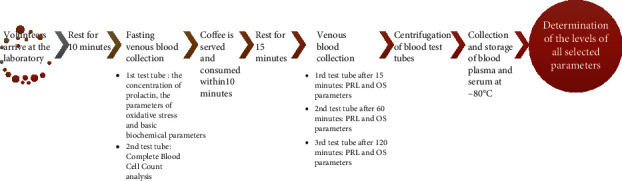
Schematic representation of experimental procedures. PRL: prolactin; OS: oxidative stress.

Venous blood was collected from the participants at four time points, as presented in Figure 2. All the participants were asked to drink one dose of such prepared coffee. For each participant, the dose of coffee was the same, regardless of body weight. In the collected biological material CBC analysis was performed using auto 5-Diff Hematology Analyzer Mindray BC-5150 (Mindray Bio-Medical Electronics Co., Ltd., Shenzhen, China). The serum concentrations of PRL, selected parameters of oxidative stress (TAS, FRAP, TOS, AOPP, T-Bil, UA, ALB and Fe), calcium, magnesium, and levels of the inflammatory marker CRP were determined using methods/tests as described below. This study was conducted according to the guidelines of the Helsinki II declaration, and the protocol was approved by the Bioethics Human Research Committee of Wroclaw Medical University (No. 862/2020 and No. 865/2021).

### 2.3. Blood Collection and Handling

Whole blood samples were collected by venipuncture from an antecubital vein into four vacutainer tubes with a clotting activator and one K_2_EDTA tube. The first sampling, between 7.00 a.m. and 9.00 a.m., concerned fasting conditions (minimum 8-hour fast) before oral coffee administration. Two tubes were used—the first with a clotting activator to obtain blood serum and the second with K_2_EDTA to Complete Blood Cell Count (CBC) analysis. The second, third and fourth collections took place 15, 60, and 120 minutes after drinking coffee (a total of 3 clotting tubes to obtain serum). According to the coffee manufacturer's (Tchibo, product reference number: tcs81037, United Kingdom) information, one dose of coffee (100% Arabica instant coffee, freeze-dried, harmoniously mild, subtle acidity) contained 72 mg of caffeine/2.4 kcal/<0.12 g of fat/<0.12 carbohydrates/<0.012 g of salt/0.36 g of proteins in 1.8 g instant coffee, and a dose of coffee drink was prepared by dissolving 1.8 g instant coffee in 200 mL hot water. Subsequently, samples were centrifuged at 1500 g for 10 min. After centrifugation, blood plasma was collected from the K_2_EDTA tube and blood serum was collected from vacutainer clotting activator tubes and stored at –80°C for future analysis.

### 2.4. Statistical Analysis

The Statistica 13.3 PL software (StatSoft Poland Sp. z o.o., Krakow, Poland) was used to analyze the results statistically. All data were tested with the Shapiro–Wilk test for normality distribution. Due to the lack of a normal distribution for examined parameters at some time points and the small size of the study groups, nonparametric tests for dependent variables were used for data analysis. In the first step, Friedman's ANOVA rank test was used to determine whether there were differences between concentrations of each parameter, depending on the time the blood sample was taken. This test is analogous to Repeated Measures ANOVA, but with the advantage of being nonparametric, and not requiring the assumptions of normality or homogeneity of variances. Friedman's ANOVA was used to determine if the consumption of caffeinated coffee significantly influences the measurements obtained in any of the four time points. In the second step, in both groups, the Wilcoxon Signed-Rank test was used to compare the differences between values at 15', 60', and 120' time points in reference to values obtained at time point 0'. The results were presented as the mean ± SD (standard deviation) and median (Me) with interquartile range (Q1–Q3) and as Me and minimum-maximum range in Figure S1 (Supplementary materials (available here)). Spearman's rank correlation was used to check the relationships between the measured parameters, separately for each time point. The strength of Spearman's rank correlations was rated based on the following classification: 0.0 ≤ |*R*| ≤ 0.2—lack of correlation; 0.2 < |*R*| ≤ 0.4—weak correlation; 0.4 < |*R*| ≤ 0.7—moderate correlation; 0.7 < |*R*| ≤ 0.9—strong correlation; and 0.9 < |*R*| ≤ 1.0—very strong correlation. A two-tailed *p* value of less than 0.05 was considered significant.

### 2.5. Assay Measurements

#### 2.5.1. Prolactin

Prolactin concentrations were determined with the commercially available ELISA test–DRG Prolactin ELISA (EIA-1291) (DRG Instruments GmbH, Marburg, Germany), according to the recommendations of the manufacturer. The linearity range was up to 200 ng/mL. A Mindray-96A reader (Mindray, Shenzhen, China) was used to measure the absorbance in this assay.

#### 2.5.2. TAS

TAS was measured using the 2,2′-azino-di-3-ethylbenzthiazoline sulfonate (ABTS)^+^ colorimetric method (Randox TAS Kit, Crumlin, United Kingdom). This method depends on the ability of antioxidants contained in the serum to inhibit the formation of ABTS^+^ from the oxidation of ABTS by metmyoglobin (a peroxidase). The concentration of TAS was analyzed using the biochemical analyzer Konelab 20i® (ThermoScientific, Vantaa, Finland) and given in mmol/L of Trolox equivalents, with the linearity of up to 2.50 mmol/L.

#### 2.5.3. FRAP

FRAP reagent was prepared *ex tempore* by mixing 300 mmol/L acetate buffer, 10 mmol/L 2,4,6-tripyridyl-s-triazine (TPTZ) in 40 mM hydrochloric acid (HCl), and 20 mmol/L aqueous solution of FeCl_3_ × 6H_2_O with proportion 10 : 1 : 1. A calibration curve was performed for the known amounts of Fe^2+^ in the solution, from 0.05 to 0.25 mmol/L Fe^2+^. 500 *μ*L of FRAP reagent was mixed with 100 *μ*L of a diluted sample (1 : 9), incubated for 5 min at 37°C, and then centrifuged at 2000 g for 10 min at room temperature. The supernatants were analyzed spectrophotometrically at 593 nm against a reagent blank using a UV/Vis spectrophotometer (UV-6300PC, VWR, Shanghai, China). The FRAP concentrations were read from the calibration curve and expressed in mmol/L.

#### 2.5.4. TOS

TOS concentrations were measured according to a method previously published by Erel [39]. In the first step, two reagents were prepared—reagent 1 was made by mixing 22.8 mg of xylenol orange and 1.636 g NaCl with 180 mL 25 mmol/L H_2_SO_4_ and 20 mL glycerol, and reagent 2 was made by mixing 19.6 mg of ferrum ammonium sulfate and 31.7 mg of o-dianisidine dihydrochloride with 10 mL 25 mmol/L H_2_SO_4_. Then, 450 *μ*L of reagent 1 and 70 *μ*L of serum were mixed, and absorbance was measured at 560 nm with a side wave of 800 nm against a reagent blank. After measurement, reagent 2 was added, and after 3 minutes of incubation at room temperature, absorbance was measured again at 560 nm with a side wave of 800 nm. The difference between absorbance measurements at the two time points was used for further calculations. A calibration curve was performed for the absorbance of an aqueous solution of perhydrol, made by perhydrol dilution with distilled water from 0 *μ*mol/L to 25 *μ*mol/L. The concentration of TOS was analyzed using a UV/Vis spectrophotometer (UV-6300PC, VWR, Shanghai, China), read from the calibration curve, and expressed in *μ*mol/L.

#### 2.5.5. OSI

Oxidative stress index was calculated as the ratio of TOS concentration to TAS concentration [42]:

(1) $$\begin{array}{c} \text{OSI}\left(\text{arbitrary units}\right)=\frac{\text{TOS }\left(\mu \text{mol}/\text{L}\right)}{\text{TAS }\left(\text{mmol}/\text{L}\right)}. \end{array}$$

#### 2.5.6. AOPP

AOPP concentrations were measured by using 1.16 mol/L potassium iodide solution (4.825 g KI in 25 mL H_2_O). Determinations were conducted in ELISA Nunc™ MaxiSorp plates. In the first step, sera were diluted by PBS in proportion 1 : 9 (200 *μ*L) and shacked for 2 minutes at 500 rotations per minute. Then, 10 *μ*L of KI was added, followed by 20 *μ*L of 100% glacial acetic acid added after 2 minutes of incubation at room temperature. A control sample was prepared simultaneously by mixing 200 *μ*L of PBS with 10 *μ*L of KI, to which, after 2 minutes of incubation at room temperature, 20 *μ*L of 100% glacial acetic acid was added. Both samples were measured at 340 nm and 600 nm using a UV/Vis spectrophotometer (Multiskan GO, Thermo Scientific). The results were expressed in *μ*mol/L of chloramine T equivalent because a calibration curve was constructed for chloramine T concentrations ranging from 0 to 80 *μ*mol/L.

#### 2.5.7. Low-Molecular-Weight Antioxidants and Iron Measurements

Concentrations of albumin, total bilirubin, uric acid, and iron were measured by colorimetric method using the biochemical analyzer Konelab 20i® (ThermoScientific, Vantaa, Finland). All procedures were performed following the manufacturers' instructions. Lower test limits were 2.00 g/dL, 0.06 mg/dL, 0.20 mg/dL, and 6.00 *μ*g/dL, respectively.

#### 2.5.8. Calcium and Magnesium Concentrations

The concentrations of calcium and magnesium were measured by using a diagnostic reagent for quantitative *in vitro* determination produced by DiaSys Diagnostic Systems (Calcium AS FS and Magnesium XL FS, DiaSys Diagnostic Systems GmbH, Holzheim, Germany). Calcium levels were measured using the Arsenazo III test according to the manufacturer's recommendations. The test measuring range was within 0.04–20.00 mg/dL. Magnesium concentrations were measured by photometric test using xylidyl blue according to the manufacturer's procedures. The test measurements range was from 0.05 to 5.00 mg/dL, and the analyzer Konelab 20i® (ThermoScientific, Vantaa, Finland) was used for determinations.

#### 2.5.9. Inflammatory Markers

To determine C-reactive protein (CRP) concentrations, commercial reagents for the immunoturbidimetric test were used (highly sensitive for CRP, U-hs, DiaSys Diagnostic Systems GmbH, Germany), and measurements were made using an automatic analyzer Konelab 20i® (ThermoScientific, Vantaa, Finland). All procedures were performed according to the recommendations of the manufacturer. The measuring range was from 0.3 mg/L up to the concentration of the highest calibrator, at least up to 350 mg/L. White blood cell count (WBC) was obtained from a Complete Blood Cell Count analysis (5-Diff Hematology Analyzer Mindray BC-5150, Mindray Bio-Medical Electronics Co., Ltd., Shenzhen, China).

## 3. Results

### 3.1. Prolactin

**Figure 3:**
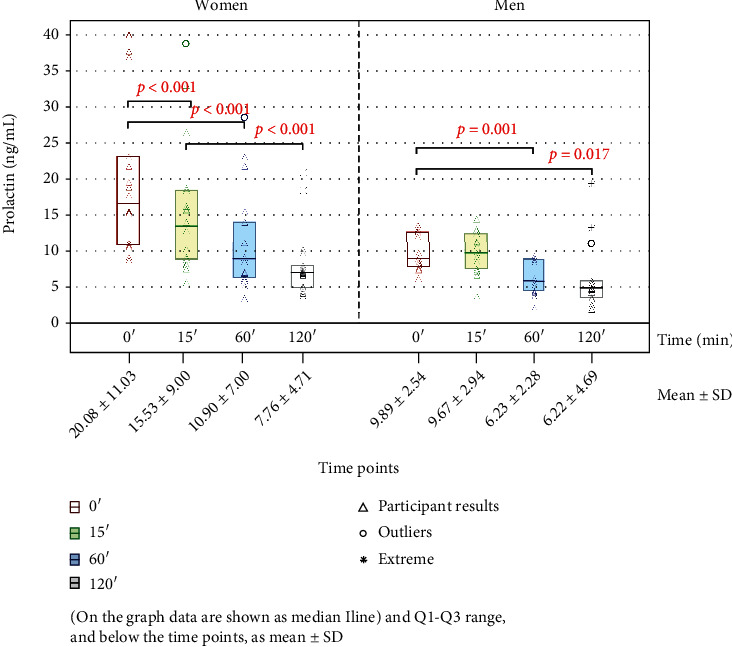
The concentration values of prolactin for women and men. SD: standard deviation; *p* value was calculated versus time point 0', and a two-tailed *p* value of less than 0.05 was considered significant. 0', 15', 60', and 120': time points of measurements.

The results of the determinations of PRL concentrations are shown in Figure 3.

Fasting PRL levels (0') were higher in women than in men (median values: 16.56 ng/mL in women, 8.85 ng/mL in men). Prolactin concentrations in women after caffeinated coffee consumption were significantly lower, at 15', 60', and 120' in comparison to time point 0' (13.43 ng/mL, 8.80 ng/mL, and 6.91 ng/mL, respectively) with a significance of *p* < 0.001 for each time point, and in men at 60' and 120' (5.74 ng/mL, *p* = 0.001, and 4.80 ng/mL, *p* = 0.017, respectively). No significant differences were found in men at time point 15' (9.59 ng/mL) when compared to time point 0'.

### 3.2. Oxidative Stress Markers

The results of the determinations of TAS, FRAP, TOS, OSI, AOPP, low-molecular-weight antioxidants (UA, T-Bil, and ALB), Fe, Ca, Mg, and inflammatory marker (CRP) concentrations are shown in Table 2, with marked significant differences in levels of investigated parameters in relation to time point 0'.

#### 3.2.1. TAC

Friedman's rank test showed that in time, after drinking coffee, there were significant differences between TAS concentrations in women (*p* = 0.044) and no differences in men. The median values at 0', 15', 60', and 120' time points for women were 1.57 mmol/L, 1.55 mmol/L, 1.56 mmol/L, and 1.57 mmol/L, respectively, and for men, they were at levels of 1.65 mmol/L, 1.70 mmol/L, 1.66 mmol/L, and 1.64 mmol/L, respectively. The Wilcoxon test indicated a lack of significant differences between TAS concentrations in 15', 60', and 120' in comparison to time point 0' in both groups. FRAP concentrations significantly differed in time for women (*p* = 0.002) and were similar for men at all time points. The median of FRAP levels at 15', 60', and 120' time points for women was 1.13 mmol/L, 1.15 mmol/L, and 1.13 mmol/L, respectively, and was significantly higher when compared to time point 0' (1.11 mmol/L), with the significance of *p* < 0.001, *p* < 0.001, and *p* = 0.028, respectively. Such differences were not observed for men (median values: 1.34 mmol/L, 1.39 mmol/L, 1.37 mmol/L, and 1.38 mmol/L at time points 0', 15', 60', and 120', respectively).

#### 3.2.2. TOS and OSI

No significant differences were found in TOS levels and OSI, neither for women nor men. In women, the median values of TOS concentrations at time points 0', 15', 60', and 120' were 1.50 *μ*mol/L, 1.57 *μ*mol/L, 1.44 *μ*mol/L, and 1.38 *μ*mol/L, respectively. In the male group, the median values for TOS levels at time points 0', 15', 60', and 120' were 1.38 *μ*mol/L, 1.71 *μ*mol/L, 1.40 *μ*mol/L, and 1.45 *μ*mol/L, respectively. The OSI values calculated for women at time points 0', 15', 60', and 120' were 0.95, 1.00, 0.93, and 0.92, respectively, and for men, they were 0.91, 1.08, 0.85, and 0.88, respectively.

#### 3.2.3. AOPP

There were no significant differences in AOPP concentrations between time points 15', 60', and 120' in reference to time point 0', neither in women (median values for 0', 15', 60', and 120': 85.65 *μ*mol/L, 84.99 *μ*mol/L, 86.31 *μ*mol/L, and 82.02 *μ*mol/L, respectively) nor in men at time points 60' and 120' (median values: 87.96 *μ*mol/L and 84.66 *μ*mol/L, respectively). Significant differences in reference to time point 0' (median value: 87.96 *μ*mol/L) were observed in men at 15' (median value: 90.61 *μ*mol/L) with significance of *p* = 0.047.

#### 3.2.4. Low-Molecular-Weight Antioxidants

The differences in uric acid concentrations were not significant at the examined time points, neither for women nor men. Median values of UA concentrations for women were 4.68 mg/dL at 0', 4.50 mg/dL at 15', 4.66 mg/dL at 60', and 4.54 mg/dL at 120', and only the concentrations at time point 120' were significantly lower when compared to these at time point 0' (*p* = 0.048). No significant differences were found in men, where the median values of UA concentrations at time points 0', 15', 60', and 120' were 5.71 mg/dL, 5.77 mg/dL, 5.63 mg/dL, and 5.70 mg/dL, respectively.

Total bilirubin concentrations were similar in women (median values at 0', 15', 60', and 120' were 0.55 mg/dL, 0.55 mg/dL, 0.57 mg/dL, and 0.54 mg/dL, respectively) while in men T-Bil concentrations significantly increased over time (*p* < 0.001) and the differences between time point 15' (median value: 0.62 mg/dL, *p* = 0.005), 60' (median value: 0.67 mg/dL, *p* < 0.001), and 120' (median value: 0.69 mg/dL, *p* < 0.001) were significant in reference to time point 0' (median value: 0.61 mg/dL).

Albumin concentrations changed over time in both women and men, with a significance of *p* = 0.006 and *p* = 0.002, respectively. Among women, the median values of ALB concentrations were 4.45 g/dL, 4.30 g/dL, 4.42 g/dL, and 4.52 g/dL, while among men the median values of ALB levels were 4.59 g/dL, 4.54 g/dL, 4.55 g/dL, and 4.59 g/dL for 0', 15', 60', and 120', respectively. Only at time point 15' in women (*p* < 0.001) and at time point 15' in men (*p* < 0.001) ALB concentrations were significantly lower in comparison to time point 0'.

### 3.3. Elements

Based on the results of Friedman's ANOVA test, we observed that the consumption of caffeinated coffee influences the concentration of serum elements, as the analyzed groups differed significantly at four time points in both women and men for Fe (*p* = 0.038 and *p* = 0.018, respectively), in women for Mg (*p* = 0.032) and in men for Ca (*p* = 0.019). The median values of concentrations of Fe at time points 0', 15', 60', and 120' in women were 101.50 *μ*g/dL, 98.50 *μ*g/dL, 108.00 *μ*g/dL, and 108.50 *μ*g/dL, respectively, and in men, they were 109.00 *μ*g/dL, 106.00 *μ*g/dL, 105.00 *μ*g/dL, and 108.00 *μ*g/dL, respectively. In men only at time points 60' and 120' were Fe levels significantly higher in reference to time point 0' with *p* = 0.023 and *p* = 0.011, respectively. For Ca concentrations in women, the median values at 15', 60', and 120' time points were 9.55 mg/dL, 9.60 mg/dL, and 9.70 mg/dL, respectively, and there were no significant differences in relation to time point 0' (9.45 mg/dL). For Mg concentrations in women, no significant differences between time point 0' and the other time points were observed (2.30 mg/dL, 2.20 mg/dL, 2.30 mg/dL, and 2.35 mg/dL, respectively). On the other hand, in men, the median values of Ca levels measured at all defined time points were 9.50 mg/dL, 9.50 mg/dL, 9.50 mg/dL, and 9.70 mg/dL, respectively, with significantly higher values observed for time point 120' (*p* = 0.018) in reference to time point 0'. Meanwhile, the following Mg levels were observed: 2.30 mg/dL, 2.30 mg/dL, 2.20 mg/dL, and 2.40 mg/dL, respectively, and did not differ significantly.

### 3.4. Inflammatory Marker

The CRP concentrations were similar for women and men and did not differ significantly between the analyzed time points, neither in women (median values: 1.33 mg/L, 1.17 mg/L, 1.25 mg/L, and 1.23 mg/L, respectively) nor in men (0.47 mg/L, 0.46 mg/L, 0.45 mg/L, and 0.42 mg/L, respectively).

### 3.5. Correlations

In Table 3, we presented the results of Spearman's rank correlations only for pairs of analyzed parameters that correlated significantly at least at three time points of measurements. The results were analyzed separately for women and men, as blood serum prolactin physiological levels significantly differ between genders. Additionally, it was documented that uric acid levels are physiologically lower in women than in men, and simultaneously UA, as a component of TAS and FRAP, influence these levels, although to a different extent.

We observed a positive moderate correlation between calcium and albumin levels at all time points in both genders, except time point 0' in men. Calcium concentrations also showed positive, moderate, and strong correlations with magnesium levels in men. In turn, for women, we found a negative moderate correlation between CRP and calcium concentrations and a negative strong correlation between CRP and albumin levels. Strong or very strong positive correlations were observed between uric acid and FRAP levels, regardless of gender. In men, we also showed a strong positive correlation between UA and TAS concentrations and between TAS and FRAP levels.

## 4. Discussion

Hyperprolactinemia can threaten many processes in the human body through hormonal disorders, but also through the induction of oxidative stress [30], which is often overlooked in assessing the effects of excess prolactin in the organism. In our study, prolactin levels in healthy participants were independent of BMI (data not shown) and were generally higher in women than in men (median values: 16.56 ng/mL and 8.85 ng/mL, respectively), which is consistent with the physiological difference between the sexes. After administration of a single dose of caffeinated coffee, a decrease in prolactin levels was observed over time, both in men and in women. Our results also showed a significant reduction in prolactin levels at different time points of PRL measurements (15', 60', and 120') in reference to time point 0', except time point 15' in men. On the other hand, the study by Kotsopoulos et al. [36], who investigated the effect of caffeine and caffeinated coffee on the concentration of prolactin among pre- and postmenopausal women (aged 25–70 years), depending on the number of cups of coffee and caffeine consumed daily, have shown that there were no significant differences in the levels of this hormone. The differences between our observations and results obtained by Kotsopoulos et al. [36] may be caused by differing time periods in which PRL levels were examined after the consumption of caffeinated beverages. While Kotsopoulos et al. [36] analyzed the long-term impact of caffeine beverages on prolactin levels, our study showed a short-term decrease in serum prolactin concentrations. Differences in the age of participants included in the study groups are another possible cause of the observed dissimilarity in PRL concentration analyzed in relation to coffee consumption between the study of Kotsopoulos et al. [36] and our investigations. Our participants were 19-29 years old, while the subjects analyzed by Kotsopoulos et al. [36] were aged 25–70. However, due to the real short-term effect of coffee ingredients on the level of prolactin, further, more extensive research is needed to draw more significant conclusions. We were the first to investigate the influence of caffeinated coffee on the level of blood serum PRL not only in women but also in men. Although all the concentrations of prolactin observed were within the reference ranges, the evident effect of caffeinated coffee and its ingredients on the reduction of PRL levels may suggest that caffeine possibly has a similar effect in the case of hyperprolactinemia, which, after confirmation in further studies, may contribute to the development of new therapeutic strategies in its treatment.

Oxidative stress plays an important role in many pathological processes that take place in the human body and is responsible for the development of a variety of diseases. Parameters of oxidative-antioxidant balance may be examined by measurements of TAS, FRAP, and TOS concentrations to estimate the overall oxidation status of the body, as well as AOPP levels and the concentrations of low-molecular-weight antioxidants such as UA, T-Bil, and ALB, which may vary during the response to oxidative stress. It is already documented that TAS levels also provide information on the relative antioxidant capacity of different coffees [6]. FRAP test is a nonspecific, redox-related colorimetric test which is related to the concentration of antioxidants present in the tested sample, and the increase in absorbance is proportional to the total ferric reducing power of the sample [43]; however, it only reflects the reducing capacity and does not identify potential antioxidants [6, 9, 12].

The presence of antioxidant compounds in coffee leads to the disappearance of free radical chromogens [6]. Most of the evidence supporting the positive role of coffee in the reduction of oxidative stress comes from *in vitro* and epidemiological studies [44–46]. In women, the determined concentrations of serum TAS slightly decreased with time after drinking caffeinated coffee, but no differences between time points 15', 60', and 120' in comparison to time point 0' were observed. In men, TAS levels slightly increased at 15' and decreased at other time points, but the differences were not significant. The results obtained for men are in accordance with the findings of Teekachunhatean et al. [47], who used the same method for TAS determination as in our study. The authors also did not find the significant differences in serum TAS levels (mean value: 1.51 mmol/L) among 11 healthy Thai male volunteers after a single dose of caffeinated coffee intake (180 mL) or measurements at time points: 10', 20', 30', 40', 60', 75', and 120' in reference to time point 0'. Significant reduction, versus baseline, in TAS levels was achieved only on the 6th and 12th day of the experiment (1.37 mmol/L and 1.39 mmol/L, respectively). Leelarungrayub et al. [48], using the same method for TAS measurements as us, examined 26 healthy men divided into three groups: subjects who consumed caffeinated coffee, subjects who consumed decaffeinated coffee, and a control group. The authors showed that there were no significant differences between the investigated groups (*p* > 0.05, mean values: 0.84 mmol/L [control], 0.98 mmol/L [decaffeinated coffee], and 1.00 mmol/L [caffeinated coffee]) in blood plasma TAS levels one hour after coffee consumption [48]. Our results for TAS concentration, analyzed in the context of caffeinated coffee intake, differ from those obtained by Leelarungrayub et al. [48], but this may be because the participants investigated by Leelarungrayub and coworkers were examined after a physical exercise test.

Another parameter that can be used to express TAC is FRAP. Our study showed that among women FRAP levels were higher over time, and differences between each time point with reference to time point 0' were significant (*p* < 0.001, *p* < 0.001, and *p* = 0.028, respectively), while among men the differences were not insignificant. Agudelo-Ochoa et al. [49] examined the impact of caffeinated coffee intake on blood plasma FRAP levels in 38 men and 37 women divided into 3 groups: control—no coffee consumption, and 2 groups that drank 1 of 2 types of coffee (400 mL/day) with different caffeine contents (188 mg/400 mL and 197 mg/400 mL) and content of other substances for 8 weeks. The authors [49] noticed that, one hour after drinking the first dose of coffee, the level of FRAP significantly increased in both groups in comparison to the baseline values, while in the control group FRAP concentrations were significantly lower. In our study, we observed similar changes in FRAP levels one hour after drinking caffeinated coffee, despite the different coffee concentrations given to our volunteers and those used in the studies of Agudelo-Ochoa et al. [49]. Moura-Nunes et al. [50] observed that blood plasma TAC levels determined using FRAP assay, measured 90 minutes after caffeinated coffee drinking (8 g of instant coffee/240 mg of caffeine/200 mL water), increased by 2.6% in a group of 10 healthy subjects (3 men and 7 women). Moreover, Metro et al. [51] reported an increase of blood plasma TAC levels for much longer than a few hours after drinking caffeine solution—it could be observed even after a week. Although the investigations of Metro et al. [51] were limited to men and different commercial tests were used to measure TAC levels, the results obtained by the authors are in accordance with our assumptions that caffeine increases TAC concentrations, which may suggest the potential influence of caffeinated coffee (polyphenols action) on total antioxidant capacity. Although in different human clinical trials coffee samples differ in their chemical composition (the variety of beans, roasting temperatures, and brewing methods), dosages, and lengths of coffee consumption examined, in general, it can be concluded that a short-term influence on oxidative-antioxidant balance parameters is observed as a result of the consumption of coffee.

The next parameters we examined were TOS levels and the OSI index. To the best of our knowledge, the effects of caffeinated coffee on TOS levels have not yet been measured. Although the OSI index is told to be a new tool for the measurement of consequences of oxidative stress [52], we are the first, to our knowledge, to use it to explore the short-term effect of caffeinated coffee on oxidative-antioxidant balance in the human body. Even though we did not observe significant differences in levels of TOS and values of OSI index between examined time points after coffee intake, both TOS levels and values of OSI index were different at each time point of measurement. The highest median value of the OSI index was observed 15 minutes after drinking caffeinated coffee; then, it stabilized at subsequent time points and the median values were comparable to the baseline value. The OSI index is the TOS/TAS ratio, which would explain these changes over time—in the initial stage after drinking caffeinated coffee, the activity of oxidative factors increases while antioxidant mechanisms are activated. Stabilization of oxidative and antioxidant mechanisms was observed 120 minutes after drinking caffeinated coffee in both sexes. We think that further extended study, using other types and/or doses of coffee, may give different results. It should also be noticed that long-term consumption of caffeinated beverages could have a potential influence on these parameters.

AOPP are products of blood plasma protein oxidation, mainly albumin. Due to the rapid response to any changes in oxidative-antioxidant balance by AOPP production, this parameter is considered suitable for measuring short-term changes in oxidative stress [53]. In our study, the changes in AOPP levels between examined time points after coffee consumption were insignificant in both groups of participants, except for time point 15' in men (*p* = 0.047). A slight decrease in AOPP levels was observed at each time point in women and at 60' and 120' in men. Nemzer et al. [54] reported that single-dose treatment with phenol-rich foods lowered blood AOPP levels by 39% in the first 60 minutes and this decrease was significant (*p* < 0.05) compared to the baseline level. AOPP concentrations returned close to baseline levels in the hour following treatment. It is very likely that our results would be in line with those obtained by Nemzer et al. [54] if we had used larger doses of coffee or a type of coffee that contained more polyphenols.

Human blood is equipped with a great number of antioxidants that can bind metal ions (for example iron and copper) and scavenge free radicals. The ability of blood plasma components to counteract oxidative stress is a useful indicator of oxidative-antioxidant status [55]. Known low-molecular-weight antioxidants include uric acid, bilirubin, and albumin—compounds that deactivate free radicals or oxidants by interacting with them [56].

UA is a powerful scavenger of blood serum oxidants, including the hydroxyl radical, singlet oxygen, ozone, and several organic and nitrogen oxidants such as peroxide radicals [56], and can therefore be considered an indirect indicator of oxidative stress [39, 57]. Moura-Nunes et al. [50] reported no significant changes in UA concentration (mean value: 3.8 mg/dL) 90 minutes from caffeinated coffee consumption (baseline: 4.0 mg/dL). On the other hand, a study conducted by Choi and Curhan [58] documented that caffeinated coffee drinking was inversely associated with UA concentration in human blood serum. Our study showed no significant changes in blood serum UA concentrations between examined time points after coffee intake, apart from time point 120' in women (*p* = 0.048), in which the median value (4.54 mg/dL) was slightly below baseline (4.68 mg/dL). Moreover, the aforementioned study conducted by Natella et al. [59] documented an increase in UA levels after the 1st and 2nd hour from drinking coffee in relation to time point 0'. The increase was attributable to the interference of phenols in UA secretion and reabsorption, as phenol-rich compounds increase UA levels [60–62]. The effect of caffeinated coffee on the concentration of UA in the blood serum is still unclear and requires further exploration, but our study, like that of Moura-Nunes et al. [50], suggests that instant coffee has no influence on this parameter. However, further research is needed to confirm or exclude this hypothesis.

In the present study, we observed strong positive correlations, importantly, at each time point, between levels of UA vs. FRAP and TAS in men and between UA vs. FRAP concentrations in women, confirming the well-known and documented positive relationships between blood plasma antioxidant activity as measured by FRAP levels and blood serum uric acid concentrations. The estimated contribution of uric acid, measured by the FRAP method, in the total antioxidant capacity is about 60%, while albumin contributes only 10%. The small participation of albumin in the results of FRAP concentration determinations is most likely related to the influence of low pH on the thiol groups of proteins required for this method [43, 63]. In turn, in the method used for the determinations of TAS concentrations, the main component is albumin (28%), while the estimated amount of uric acid is approx. 19% [63]. Physiologically, men have a higher concentration of uric acid than women, and in our investigations, we also found significantly higher concentrations of UA in the group of men (*p* = 0.002; data not shown). This is also reflected by lower serum FRAP concentration in women compared to men (*p* = 0.002; data not shown), as also documented by Brock et al. [64]. The above finding may indicate a higher contribution of uric acid in the total antioxidant capacity of blood serum in men than in women. The mechanisms of homeostatic control are very complex and require many components. Maintaining oxidative-antioxidant balance is particularly important, as oxidative stress is believed to induce many diseases. Our study indicates that these mechanisms may differ by gender, which most likely explains the presence of correlations between TAS and FRAP levels in men and not in women. On the other hand, when we took the whole group of participants, without gender differentiation, we also observed the significant correlations between TAS and FRAP levels at each time point (data not shown). A study by Erel [65] reported the presence of significant correlations between FRAP and TAS levels (*R* = 0.847, *p* < 0.0001), although Cao et al. [63] did not observe such a relationship. However, their study group consisted of 31 women and only 14 men.

Another examined parameter was T-Bil. *In vitro*, bilirubin is a scavenger of peroxyl radicals and singlet oxygen. However, whether it also fulfills this function *in vivo* remains unclear. It should also be mentioned that bilirubin is capable of forming singlet oxygen in the presence of light [56]. Moura-Nunes et al. [50] observed no significant changes in T-Bil concentrations after 90 minutes from caffeinated coffee consumption (mean value: 0.60 mg/dL) in comparison to baseline (mean value: 0.60 mg/dL). Regular consumption of caffeinated coffee contributes to the reduction of total bilirubin in human blood serum [66, 67]. Although our results were similar in the group of women at all examined time points, median values of total bilirubin concentrations among men increased slightly over the time (from 0.61 mg/dL to 0.69 mg/dL).

Albumin is one of the main components of the antioxidant defense system, the primary target of blood plasma proteins under oxidative stress, and works through its multiple binding sites and free radical scavenging properties [68]. In the present study, albumin concentrations were significantly lower both in the group of women and men at time point 15' (median value: 4.30 g/dL and 4.54 g/dL, respectively) in reference to time point 0' (4.45 g/dL and 4.59 g/dL, respectively). No other significant changes were observed, which corresponds to the results obtained by Moura-Nunes et al. [50], who observed no significant changes in albumin concentrations (mean value: 4.50 g/dL) 90 minutes from caffeinated coffee consumption (baseline mean value: 4.20 g/dL). The decrease in albumin concentration shortly after drinking caffeinated coffee may be explained by damage to blood plasma proteins by the drink's ingredients, as described by some authors [69, 70].

Oxidative stress has been speculated to relate to the disturbance of ion homeostasis in the human body due to the modification of proteins and opening of ion channels [71, 72]. Iron is a microelement responsible for oxygen transport, energy metabolism, and electron transfer. It is a cofactor of many enzymes and therefore very important in the fight against free radicals when present in physiological amounts in the body. The reported increase of iron levels in the body may be due to iron's presence in coffee [73]. On the other hand, coffee can inhibit iron absorption by phenolic compounds, which can hinder the absorption of this element. However, we did not observe the presence of significant differences in iron levels between the analyzed time points in women and saw a slight, but significant, increase of iron concentrations in men at time points 60' and 120' in comparison to baseline time point 0' (*p* = 0.023 and *p* = 0.011, respectively).

*In vitro* studies suggest a relationship between the occurrence of oxidative stress and the increased concentration of calcium in cell cytoplasm due to disturbed thiol homeostasis increasing the flow of calcium from the endoplasmic reticulum through cell membranes to the cytosol of the cell [71, 72]. In an extensive review by Olechno et al. [74], instant coffee was reported to contain high amounts of calcium, but our results do not support this statement—a significant increase in calcium concentration was observed only in men at time point 120'. Analyses of NHANES 2011–2016 data by Rehm et al. [75] have also reported that there was no association between coffee consumption and calcium supply. In our studies, we observed the presence of strong positive correlations between Ca vs. ALB concentrations in both women and men, except at point 0' in men. A positive correlation between these parameters was shown earlier by Rajaraman et al. [76]. The observed correlations result from the fact that albumin binds calcium, and this ability of ALB is necessary for the maintenance of serum calcium levels. We also observed a positive correlation between Ca vs. Mg levels in men at each time point, which may be explained by the participation of magnesium in the conversion of the inactive form of vitamin D to the active form, which increases the body's ability to absorb calcium [77]. A possible cause of such correlations, which appeared in men only, is higher blood serum vitamin D levels in men than in women [78], due to which the effect of magnesium levels on calcium levels is more noticeable in males.

The results of some studies have confirmed the positive influence of magnesium in combating the effects of oxidative stress [79] and state that magnesium deficiency is accompanied by increased levels of oxidative stress markers, such as products of lipids, proteins, and DNA oxidative modification [80]. Olechno et al. [74] showed that instant coffee has a lower magnesium content than other types of coffee. Analyses of NHANES 2011–2016 Data made by Rehm et al. [75] showed that the supply of magnesium increases with the amount of caffeinated coffee consumed (*p* < 0.001), but in the present study, no significant changes in magnesium levels between analyzed time points were observed, neither in women nor men.

Inflammation is accompanied by increased oxidative stress, and the reduction of expression of inflammation-related biomarkers as a result of coffee consumption had an antioxidant effect that accompanied the reduction of inflammation [6]. Coffee contains a variety of aforementioned bioactive compounds with anti-inflammatory and antioxidative properties (Figure 1), which may reduce blood serum CRP levels. In a long-term study on the influence of coffee intake on the concentration of serum CRP, Hang et al. [81] reported that CRP levels were significantly lower (about 16.6%) among people consuming more than 4 cups of caffeinated coffee/day in reference to those who were not. Moreover, this relationship was stronger in women than in men. Similar results were obtained in many studies [82–85]; however, the results of our study did not support the hypothesis that short-term consumption of caffeinated coffee decreases blood serum CRP levels, as no significant changes between examined time points after caffeinated coffee consumption were observed. On the other hand, we observed the presence of negative correlations between CRP and ALB concentrations in women. This is most likely related to the role of these proteins in inflammation and the balance between their expression: albumin is a negative (decrease in concentration) and CRP is a positive (increase in concentration) acute phase protein [86]. The above correlations did not occur in men, probably due to the effect of the amount and distribution of body fat on CRP level, which is greater in women than in men [87], and a faster decrease in the levels of albumin in women than in men [88]. We also showed a negative correlation between Ca and CRP levels in women only, which seems to be associated with negative correlations observed between CRP vs. ALB concentrations and positive correlations between Ca vs. ALB levels. This, in turn, may be associated with the abovementioned greater influence of body fat on CRP level in female participants.

## 5. Conclusions

**Figure 4:**
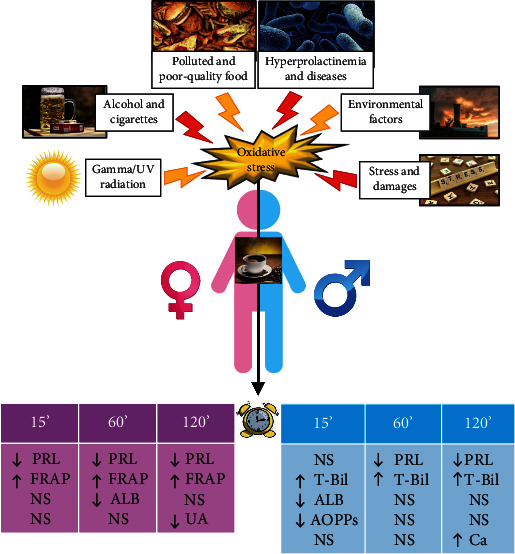
Scheme of interactions between oxidative stress development and levels of serum oxidative stress parameters in relation to time points of measurement after caffeinated coffee consumption. ALB: albumin; Ca: calcium; FRAP: ferric reducing antioxidant power; PRL: prolactin; T-Bil: total bilirubin; UA: uric acid. Versus time point 0': NS: not significant; ↑: significantly increased; ↓: significantly decreased.

We examined the short-term effect of caffeinated coffee consumption on the concentration of prolactin, selected parameters of oxidative stress, and some elements in blood sera of young healthy adults. Currently, there is very little data available on the effects of the consumption of caffeinated beverages on the body's hormone balance, especially on sex hormones. To the best of our knowledge, to date, there have been no studies analyzing the short-term effects of caffeine on values of the sex hormone PRL in both women and men. Due to the potential pharmaceutical use of caffeinated coffee ingredients, we hypothesized that caffeine may have therapeutic potential in hyperprolactinemia treatment, due to an observed reduction in the concentration of this parameter within 2 hours after consuming a caffeinated coffee. Although our results seem to be promising, further studies are needed to determine if this effect is of biological importance, especially in people struggling with hyperprolactinemia. The results of this study could help not only to understand the role of diet in maintaining the body's hormonal balance but also provide opportunities to verify the potential utility of coffee ingredients to create new therapeutic strategies and guide future research on this subject. The results obtained for the parameters of the oxidative-antioxidant balance suggest the presence of a relationship between the expression of caffeinated coffee components and a decrease in oxidative stress in the human body. The parameters of oxidative-antioxidant balance we investigated were TAS, FRAP, TOS, OSI index, AOPP, iron, calcium, magnesium, and low molecular weight antioxidants such as uric acid, total bilirubin, and albumin. We observed that caffeinated coffee increased the concentrations of some of the characteristic oxidative stress markers shortly after consumption. Although the antioxidant response after caffeinated coffee intake was not as high as expected, we may notice the potential positive role of coffee in the reduction and/or elimination of oxidative stress. An interesting direction for further, more detailed research, also based on the analysis of a wider spectrum of oxidative stress parameters, would be to check whether the ingredients of caffeinated coffee may have an important use in the prevention and treatment of the effects of oxidative stress. We hope that our findings will be helpful in developing new strategies for treating hyperprolactinemia and will contribute to increasing the significance of research on preventing the negative effects of oxidative stress. A scheme showing the interrelationships between oxidative stress development and the analyzed parameters, summarizing the results of our research, is presented in Figure 4.

## Figures and Tables

**Table 1:** General characteristics of the study groups.

Parameters	Gender	
	Women *N* = 18	Men *N* = 15
	Mean ± SD	Mean ± SD
Age (years)	22.17 ± 1.86	23.80 ± 2.60
Height (m)	1.68 ± 0.07	1.82 ± 0.05
Body mass (kg)	64.87 ± 15.38	75.02 ± 11.11
BMI (kg/m^2^)	22.85 ± 4.54	22.52 ± 2.62
WHR	0.77 ± 0.06	0.84 ± 0.11
WBC (G/L)	6.75 ± 1.86	5.63 ± 1.08
Caffeine consumption (% of people who consume caffeine)	100%	100%
The mean frequency of consumption of caffeinated beverages (times/day)	2.06 ± 1.47	2.27 ± 1.03
The frequency of consumption of caffeinated coffee (cups/day)	1.50 ± 0.86	1.93 ± 0.96
The type of consumed coffee (number of volunteers)		
Instant	3	3
Brewed coffee	7	6
Espresso coffee	7	6
Other	1	0

BMI: body mass index (body mass (kg)/height (m^2^)); WHR: waist-hip ratio (waist circumference/hip circumference); WBC: white blood cell count; SD: standard deviation; *N*: number of participants.

**Table 2:** The concentrations of oxidative-antioxidant balance parameters, elements, and inflammatory marker.

	Women *N* = 18				Men *N* = 15			
	0'	15'	60'	120'	0'	15'	60'	120'
	Mean ± SD Median (Q1-Q3)	Mean ± SD Median (Q1-Q3)	Mean ± SD Median (Q1-Q3)	Mean ± SD Median (Q1-Q3)	Mean ± SD Median (Q1-Q3)	Mean ± SD Median (Q1-Q3)	Mean ± SD Median (Q1-Q3)	Mean ± SD Median (Q1-Q3)
TAC								
TAS (mmol/L)	1.58 ± 0.08 1.57 (1.52–1.65)	1.55 ± 0.07 1.55 (1.51–1.60)	1.59 ± 0.08 1.56 (1.54–1.65)	1.58 ± 0.08 1.57 (1.50–1.62)	1.65 ± 0.08 1.65 (1.58–1.74)	1.68 ± 0.13 1.70 (1.61–1.74)	1.66 ± 0.08 1.67 (1.59–1.72)	1.66 ± 0.08 1.64 (1.61–1.75)
FRAP (mmol/L)	1.11 ± 0.11 1.11 (1.04–1.20)	1.14 ± 0.11 1.13 (1.10–1.22) *p* < 0.001	1.14 ± 0.10 1.15 (1.09–1.20) *p* < 0.001	1.13 ± 0.10 1.13 (1.08–1.19) *p* = 0.028	1.31 ± 0.19 1.34 (1.17–1.44)	1.33 ± 0.16 1.39 (1.18–1.43)	1.31 ± 0.17 1.37 (1.18–1.43)	1.33 ± 0.16 1.38 (1.20–1.45)
TOS and OSI								
TOS (*μ*mol/L)	1.72 ± 0.99 1.50 (1.07–2.16)	1.74 ± 0.96 1.57 (1.10–2.14)	2.04 ± 1.76 1.44 (0.89–3.04)	1.99 ± 2.27 1.38 (0.68–2.53)	1.94 ± 1.17 1.38 (1.20–2.38)	1.86 ± 1.01 1.71 (0.75–2.66)	1.55 ± 0.60 1.40 (1.07–1.77)	1.45 ± 0.68 1.45 (0.94–2.12)
OSI (arbitrary units)	1.10 ± 0.66 0.95 (0.68–1.42)	1.12 ± 0.62 1.00 (0.72–1.43)	1.27 ± 1.03 0.93 (0.58–2.00)	1.24 ± 1.32 0.92 (0.43–1.65)	1.17 ± 0.70 0.91 (0.68–1.44)	1.13 ± 0.64 1.08 (0.44–1.80)	0.93 ± 0.35 0.85 (0.64–1.07)	0.88 ± 0.43 0.88 (0.59–1.25)
AOPP								
AOPP (*μ*mol/L)	95.46 ± 30.15 85.65 (77.39–110.45)	87.49 ± 14.16 85.00 (78.71–97.22)	87.08 ± 16.76 86.31 (75.40–99.89)	87.56 ± 23.74 82.02 (70.77–91.93)	106.83 ± 39.79 87.97 (80.69–140.20)	97.00 ± 32.38 90.61 (74.08–113.09) *p* = 0.047	101.98 ± 35.05 87.97 (76.73–122.35)	98.72 ± 36.40 84.67 (75.40–108.46)
Low-molecular-weight antioxidants								
UA (mg/dL)	4.58 ± 0.71 4.68 (3.97–4.96)	4.54 ± 0.70 4.50 (3.94–4.93)	4.50 ± 0.67 4.66 (3.90–4.94)	4.48 ± 0.59 4.54 (3.97–4.85) *p* = 0.048	5.70 ± 1.16 5.71 (5.03–6.53)	5.74 ± 0.96 5.77 (5.00–6.45)	5.74 ± 1.01 5.63 (4.98–6.60)	5.76 ± 0.93 5.70 (5.00–6.61)
T-Bil (mg/dL)	0.52 ± 0.21 0.55 (0.42–0.60)	0.53 ± 0.22 0.55 (0.41–0.60)	0.56 ± 0.27 0.57 (0.40–0.64)	0.55 ± 0.27 0.54 (0.46–0.64)	0.78 ± 0.51 0.61 (0.42–1.17)	0.82 ± 0.53 0.62 (0.45–1.21) *p* = 0.005	0.87 ± 0.53 0.67 (0.46–1.24) *p* < 0.001	0.89 ± 0.56 0.69 (0.47–1.24) *p* < 0.001
ALB (g/dL)	4.59 ± 0.46 4.45 (4.24–4.67)	4.42 ± 0.30 4.30 (4.24–4.57) *p* < 0.001	4.65 ± 0.50 4.42 (4.35–5.19)	4.67 ± 0.53 4.52 (4.32–4.70)	4.72 ± 0.38 4.59 (4.50–5.13)	4.50 ± 0.18 4.54 (4.41–4.65) *p* < 0.001	4.55 ± 0.28 4.55 (4.35–4.70)	4.78 ± 0.44 4.59 (4.39–5.27)
Elements								
Fe (*μ*g/dL)	96.39 ± 41.15 101.50 (68.00–125.00)	95.06 ± 41.33 98.50 (67.00–124.00)	100.78 ± 45.73 108.00 (63.00–132.00)	101.44 ± 47.71 108.50 (63.00–132.00)	114.47 ± 43.59 109.00 (87.00–127.00)	114.80 ± 43.59 106.00 (86.00–126.00)	118.27 ± 46.04 105.00 (88.00–135.00) *p* = 0.023	121.40 ± 45.13 108.00 (90.00–140.00) *p* = 0.011
Ca (mg/dL)	9.48 ± 0.47 9.45 (9.10–9.60)	9.51 ± 0.39 9.55 (9.20–9.70)	9.63 ± 0.40 9.60 (9.40–9.90)	9.63 ± 0.41 9.70 (9.40–9.90)	9.49 ± 0.36 9.50 (9.20–9.80)	9.49 ± 0.40 9.50 (9.20–9.70)	9.59 ± 0.44 9.50 (9.20–9.90)	9.66 ± 0.37 9.70 (9.30–9.90) *p* = 0.018
Mg (mg/dL)	2.29 ± 0.14 2.30 (2.20–2.40)	2.25 ± 0.15 2.20 (2.20–2.30)	2.30 ± 0.11 2.30 (2.20–2.40)	2.34 ± 0.15 2.35 (2.20–2.40)	2.33 ± 0.15 2.30 (2.20–2.40)	2.31 ± 0.16 2.30 (2.20–2.40)	2.27 ± 0.18 2.20 (2.20–2.40)	2.33 ± 0.20 2.40 (2.20–2.50)
Inflammatory marker								
CRP (mg/L)	2.06 ± 2.34 1.33 (0.44–2.35)	2.01 ± 2.28 1.17 (0.45–2.41)	2.07 ± 2.42 1.25 (0.29–2.40)	2.09 ± 2.45 1.23 (0.36–2.43)	0.58 ± 0.62 0.47 (0.15–0.62)	0.58 ± 0.60 0.46 (0.18–0.61)	0.58 ± 0.62 0.45 (0.19–0.55)	0.59 ± 0.62 0.42 (0.22–0.68)

The Wilcoxon test was used to check the differences between time point 0' and other analyzed time points. A two-tailed *p* value of less than 0.05 was considered significant. *p*: significant differences versus time point 0'. ALB: albumin; AOPP: advanced protein oxidation products; Ca: calcium; CRP: C-reactive protein; SD: standard deviation; Fe: iron; FRAP: ferric reducing antioxidant power; Mg: magnesium; OSI: oxidative stress index (TOS/TAS); TAC: total antioxidant capacity; TAS: total antioxidant status; TOS: total oxidant status; T-Bil: bilirubin; UA: uric acid.

**Table 3:** The significant correlations between concentrations of determined parameters.

Parameters compared	0'		15'		60'		120'	
	*R*	*p*	*R*	*p*	*R*	*p*	*R*	*p*
Women								
Ca vs. ALB	0.586	0.010	0.667	0.002	0.612	0.007	0.646	0.004
Ca vs. CRP	-0.559	0.016	-0.541	0.020	-0.552	0.017	-0.516	0.028
CRP vs. ALB	-0.717	<0.001	-0.735	<0.001	-0.717	0.001	-0.664	0.003
FRAP vs. UA	0.830	<0.001	0.904	<0.001	0.884	<0.001	0.687	0.002
Men								
Ca vs. ALB	0.503	0.056	0.560	0.030	0.704	0.003	0.558	0.031
Ca vs. Mg	0.636	0.011	0.548	0.034	0.710	0.003	0.757	0.001
FRAP vs. UA	0.961	<0.001	0.946	<0.001	0.968	<0.001	0.957	<0.001
TAS vs. UA	0.893	<0.001	0.821	<0.001	0.862	<0.001	0.811	<0.001
FRAP vs. TAS	0.859	<0.001	0.728	0.002	0.803	<0.001	0.706	0.003

A two-tailed *p* value of less than 0.05 was considered significant. ALB: albumin; Ca: calcium; CRP: C-reactive protein; FRAP: ferric reducing antioxidant power; Mg: magnesium; R: correlation coefficient; TAS: total antioxidant status; UA: uric acid.

## Data Availability

The data presented in this study are available upon reasonable request from the corresponding author.

## Supplementary Materials

Supplementary Materials Figure S1: The concentration values of selected parameters in women and men.
