# Supplementary material for: The Examination of the Influence of Caffeinated Coffee Consumption on the Concentrations of Serum Prolactin and Selected Parameters of the Oxidative-Antioxidant Balance in Young Adults: A Preliminary Report
Source: Oxid Med Cell Longev. 2022 Jul 25;2022:1735204. doi: 10.1155/2022/1735204 (PMC9343215; doi:10.1155/2022/1735204)
Supplement: Supplementary Materials — Figure S1: The concentration values of selected parameters in women and men. [file 1735204.f1.docx]

**Oxidative Medicine and Cellular Longevity**

**The examination of the influence of caffeinated coffee consumption on the concentrations of serum prolactin and selected parameters of the oxidative–antioxidant balance in young adults – preliminary report**

Kamil Rodak ^1^, Izabela Kokot ^1^, Aleksandra Kryla ^1^, Ewa Maria Kratz ^1*^

^1^ Department of Laboratory Diagnostics, Division of Laboratory Diagnostics, Faculty of Pharmacy, Wroclaw Medical University, Borowska Street 211A, 50-556 Wroclaw, Poland; krodak98@icloud.com (K.R.); izabela.kokot@umw.edu.pl (I.K.); alekskryla@gmail.com (A.K.); ewa.kratz@umw.edu.pl (E.M.K.).

*Correspondence should be addressed to Ewa M. Kratz: ewa.kratz@umw.edu.pl

**Figure S1.** The concentration values of selected parameters in women and men. *p* – significant differences versus time point 0’. A two-tailed *p*-value of less than 0.05 was considered significant. AOPP – advanced protein oxidation products; FRAP – ferric reducing antioxidant power; T-Bil – total bilirubin.
